# Incidence and factors associated with serosal and soft tissue enhancement after cardiac catheterization in infants

**DOI:** 10.1007/s00247-025-06343-x

**Published:** 2025-08-02

**Authors:** Akhil Dhamija, Joshua D. Wermers, Sarosh P. Batlivala, Yinan Li, Bin Zhang, Alexander J. Towbin

**Affiliations:** 1 Department of Radiology, The Ohio State University Wexner Medical Center, Columbus, USA; 2 Department of Radiology, Phoenix Childrens Hospital, Phoenix, USA; 3 Heart Institute, Cincinnati Children’s Hospital; Division of Pediatric Cardiology, University of Cincinnati College of Medicine, 3333 Burnet Ave, MLC 5031, Cincinnati, OH 45229 USA; 4 Department of Radiology, Cincinnati Children’s Hospital; Department of Radiology, University of Cincinnati, Cincinnati, USA; 5 Division of Biostatistics and Epidemiology, Cincinnati Children’s Hospital; Department of Pediatric, University of Cincinnati, Cincinnati, USA

**Keywords:** Serosal and soft tissue enhancement, Cardiac catheterization, Pneumoperitoneum

## Abstract

**Background:**

Diffuse serosal and soft tissue enhancement is a rare imaging phenomenon observed in infants following cardiac catheterization. The appearance of serosal and soft tissue enhancement can mimic pneumoperitoneum, potentially leading to misdiagnosis and unnecessary diagnostic procedures. While serosal and soft tissue enhancement has been documented in case reports, no studies have systematically evaluated the risk factors associated with its development.

**Objective:**

To determine the frequency of serosal and soft tissue enhancement in infants following cardiac catheterization and identify clinical and imaging factors associated with this phenomenon.

**Materials and methods:**

This retrospective study analyzed infants who underwent cardiac catheterization at our institution between January 2010 and September 2019. Abdominal radiographs obtained within 2 days of the procedure were independently reviewed by three pediatric radiologists for the presence of serosal and soft tissue enhancement. Clinical data, including contrast dose, renal function, and cardiac physiology, were extracted from the electronic medical record. Statistical analysis, including *t*-tests and logistic regression, was performed to identify factors associated with serosal and soft tissue enhancement, with inter-observer reliability assessed using the Fleiss kappa test.

**Results:**

Among 1,796 infants who underwent cardiac catheterization, 294 had follow-up abdominal radiographs. Serosal and soft tissue enhancement was identified as present by all three radiologists in 21 patients (7.1%). Significant factors associated with serosal and soft tissue enhancement included lower pre- and post-catheterization creatinine levels (pre- 0.36 ± 0.17 vs 0.46 ± 0.30 mg/dL; *P*=0.043; post- 0.35 ± 0.11 vs 0.45 ± 0.29 mg/dL; *P*=0.009), higher contrast volume (31.8 ± 21.4 vs 21.0 ± 18.1 mL; *P*=0.013), and higher contrast volume per body surface area (123.0 ± 69.2 vs 80.8 ± 56.2 mL/m^2; *P*=0.002). Serosal and soft tissue enhancement occurred more frequently in patients with bi-ventricular cardiac physiology (125/294; 42.5% compared to 169/294; 57.5%).

**Conclusion:**

Serosal and soft tissue enhancement occurs in a small proportion of neonates following cardiac catheterization and is associated with higher contrast dosages and body surface area-adjusted contrast volumes. Awareness of serosal and soft tissue enhancement among pediatric radiologists is helpful to avoid misdiagnosis of pneumoperitoneum.

**Graphical Abstract:**

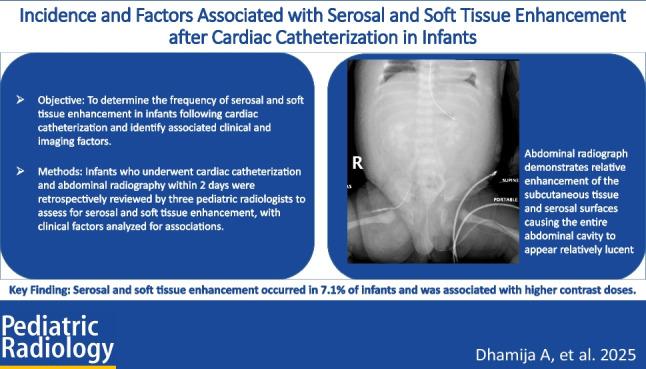

## Introduction

Diffuse serosal and soft tissue enhancement is an imaging phenomenon where the peritoneum, pericardium, and soft tissues of the torso, skin, and abdominal wall exhibit contrast enhancement after a cardiac catheterization procedure [1, 2]. It is a finding only seen in infants and, therefore, only a limited number of case reports exist [1, 2]. These reports suggest that the phenomenon may be linked to the lymphatic uptake of contrast agents. It is believed that this lymphatic uptake occurs due to extracellular fluid redistribution induced by elevated hydrostatic pressure in patients experiencing volume overload [1, 2]. The increased attenuation of the surrounding soft tissues can make the liver and spleen appear relatively lucent and may mimic pneumoperitoneum [1, 2]. Thus, the finding creates a risk of misdiagnosis, potentially leading to unnecessary further medical imaging and/or intervention.

There are currently no studies evaluating the risk factors for developing serosal and soft tissue enhancement after cardiac catheterization. Thus, the purpose of this study is to determine the frequency of serosal and soft tissue enhancement in newborns following cardiac catheterization and to determine the clinical and imaging factors associated with this condition.

## Methods

This retrospective study was approved by the institutional review board and complied with the Health Insurance Portability and Accountability Act. Patients under 1 year of age who had undergone cardiac catheterization at our institution between January 2010 and September 2019 were identified from a cardiology database. The radiology picture archiving and communication system (PACS; Merge PACS, Merative, Ann Arbor, MI) was used to identify all imaging studies performed on these patients. We included patients with available abdominal radiographs performed within 2 days of cardiac catheterization.

**Fig. 1:**
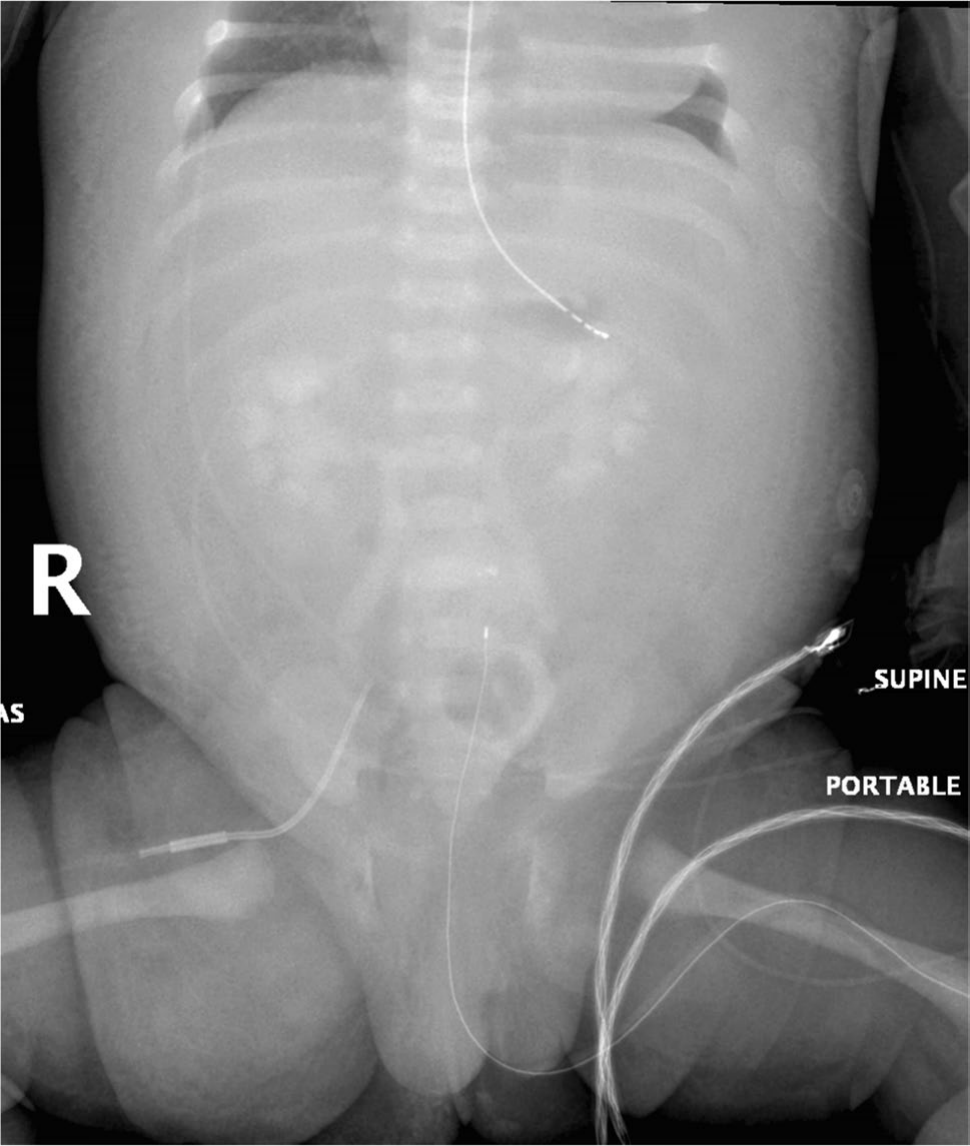
Abdominal radiograph of a 7-day-old girl with double outlet right ventricle and total anomalous pulmonary venous return who underwent cardiac catheterization 84 min prior. The radiograph demonstrates relative enhancement of the subcutaneous tissue and serosal surfaces, causing the entire abdominal cavity to appear relatively lucent

**Fig. 2:**
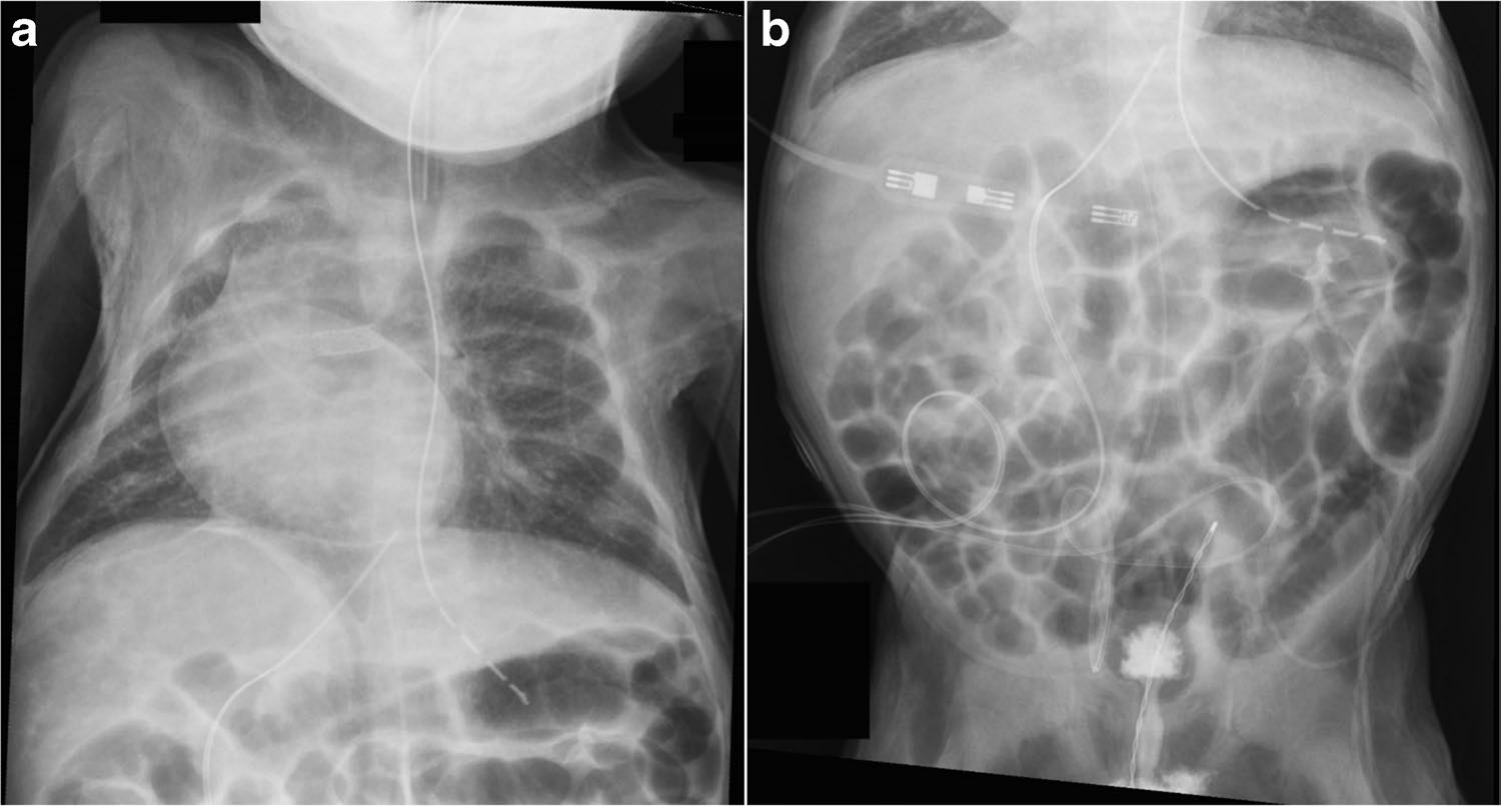
**a** Chest radiograph of a 6-day-old boy obtained 217 min after stent placement for a patent ductus arteriosus shows increased density in the subcutaneous soft tissues and the pericardium. The increased density causes the liver, heart, and some subcutaneous fat to appear more lucent than normal. This patient was misdiagnosed with pneumoperitoneum. **b** Subsequent abdominal radiograph obtained 131 min later shows increased density of the subcutaneous tissues and relative lucency of the liver. A cross-table lateral radiograph (*not shown*) confirmed that pneumoperitoneum was not present

The abdominal radiographs were independently evaluated for the presence of serosal and soft tissue enhancement by three pediatric radiologists with 1, 3, and 13 years of experience in pediatric imaging, respectively. Criteria for the presence of serosal and soft tissue enhancement on abdominal radiographs included the perceived appearance of one or more of the following findings: enhancement of the soft tissues of the abdominal wall, torso, and/or extremities; enhancement of the peritoneum or pericardium; and/or non-air lucency over the liver or spleen (Figs. 1 and 2). Reviewers met prior to their assessment of the images and agreed upon the trial definition and reviewed case examples. During review, the radiologists were blinded to the radiology report, their colleagues’ concurrent evaluations, and all clinical parameters, including contrast dose and renal function. However, they were able to view any pre-catheterization radiographs.

The electronic medical record system (Epic Systems, Verona, WI) was reviewed and demographic, laboratory, and clinical information, including contrast dose of ioversol 350 (Optiray 350; Guerbet, Villepinte, France), body surface area, renal function tests, cardiac ejection fraction, and underlying cardiac diagnosis, was abstracted. A cardiologist reviewed all cardiac catheterization reports and categorized cardiac function as either normal or abnormal. In patients with bi-ventricular physiology, the right ventricular function was assessed, as this ventricle receives systemic venous return. In patients with single ventricle physiology, the function of the single functional ventricle was evaluated. If dysfunction was present, it was further classified as mild, moderate, or severe. Grading of ventricular function was based on the formal clinical interpretation of a board-certified pediatric cardiologist with subspecialty imaging training. The frequency of serosal and soft tissue enhancement was compared between patients with normal function and those with any degree of dysfunction. Additional analysis evaluated whether the frequency of serosal and soft tissue enhancement increased with the severity of dysfunction.

Finally, the original radiology reports for the abdominal radiographs and any preceding chest radiographs were reviewed to determine whether any patients with serosal and soft tissue enhancement were misdiagnosed with pneumoperitoneum.

Statistical analysis performed via SAS version 9.4 (SAS Institute, Cary, NC) utilized a two-sample *t*-test or Wilcoxon rank sum test and univariate logistic regression analysis to determine clinical variables that correlate with serosal and soft tissue enhancement. The data normality was checked using the Shapiro-Wilk test. A wide variety of demographic and laboratory variables were included, such as creatinine levels, body surface area (BSA), contrast volume per BSA, height, weight, sex, and age. Percentages were compared using a chi-squared test. Pearson’s correlation coefficient was used to assess the correlation between pre-catheterization creatinine values and contrast dosage per BSA. Inter-observer reliability calculations were also performed using the Fleiss kappa test. *P*-values <0.05 were considered significant.

## Results

In total, 1,796 patients under 1 year of age who had undergone cardiac catheterization were identified via the cardiology database. Two hundred ninety-four patients (182 male/112 female; mean age 123 days) had an abdominal radiograph within 2 days of catheterization and were included in the study.

Reviewer 1 identified serosal and soft tissue enhancement in 44 patients (15.0%), reviewer 2 in 41 patients (13.9%), and reviewer 3 in 108 patients (36.7%). The Fleiss kappa test was applied to evaluate the level of inter-observer agreement among the three reviewers. The test produced a kappa score of 0.30 (95% CI (0.23, 0.37)), indicating fair agreement among the reviewers, which suggests agreement that was better than chance, though not particularly strong. While the number of patients identified with serosal and soft tissue enhancement was similar for reviewers 1 and 2, their agreement was not markedly better. The kappa (95%) was 0.46 (0.32, 0.61) for reviewers 1 and 2; 0.36 (0.26, 0.47) for reviewers 1 and 3; and 0.18 (0.08, 0.29) for reviewers 2 and 3. Data for individual reviewers is included in Table 1.

**Table 1:** Frequency of serosal and soft tissue enhancement by reviewer

	Serosal and soft tissue enhancement present (%)	Serosal and soft tissue enhancement absent (%)
Reviewer 1	44 (15)	250 (85)
Reviewer 2	41 (13.9)	253 (86.1)
Reviewer 3	108 (36.7)	186 (63.3)
At least two reviewers agree on the presence or absence of serosal and soft tissue enhancement (*n*=294)	45 (15.3)	249 (84.7)
All three reviewers agree on the presence or absence of serosal and soft tissue enhancement (*n*=188)	21 (7.1)	167 (56.8)

The presence of serosal and soft tissue enhancement was identified by at least two reviewers in 45 patients (15.3%) and by all three reviewers in 21 patients (7.1%). Serosal and soft tissue enhancement was considered to not be present by all reviewers in 167 patients (56.8%).

When reviewing the clinical reports, pneumoperitoneum was not described in any patient on the abdominal radiograph. However, in one patient, pneumoperitoneum was diagnosed on a preceding chest radiograph.

### Variable associations

Because there were differences in the frequency of diagnosis of serosal and soft tissue enhancement between reviewers, sub-analysis was only performed on the cohort of patients where all three reviewers agreed that serosal and soft tissue enhancement was present or absent (*n*=188).

Multiple clinical and laboratory variables were assessed to determine if they were associated with the diagnosis of serosal and soft tissue enhancement (Table 2). Of the 21 patients who were diagnosed with serosal and soft tissue enhancement by all three reviewers, creatinine values were present for 19 (90.5%) patients within 24 h before catheterization and 18 (85.7%) patients within 24 h after catheterization. Compared with the non-serosal and soft tissue enhancement cohort (*n*=167), 133 (79.6%; *P*-value=0.38) patients had a creatinine value within 24 h before the catheterization and 139 (83.2%; *P*-value=1) within 24 h after catheterization. Those with serosal and soft tissue enhancement had a significantly lower creatinine value pre- (0.36±0.17 vs 0.46±0.30 mg/dL; *P*-value=0.04) and post-catheterization (0.35±0.11 vs 0.45±0.29 mg/dL; *P*-value=0.009). However, there was no significant difference in the mean calculated GFR between patients with and without serosal and soft tissue enhancement at either time-point (73.1±30.9 vs 71.2±42.4 mL/min; *P*=0.85 pre-catheterization, and 68.5±22.3 vs 70.9±40.8 mL/min; *P*-value=0.71 post-catheterization).

**Table 2:** Association of different clinical parameters and a unanimous consensus diagnosis of serosal and soft tissue enhancement

	Serosal and soft tissue enhancement present (median (IQR) or mean ± SD)	Serosal and soft tissue enhancement absent (median (IQR) or mean ± SD)	*P*-value
Age at catheterization (days)	78 (15, 116) *n*=21	43 (2, 120) *n*=167	0.178
Height (cm)	53.2 ± 5.6 *n*=21	52.9 ± 7.5 *n*=156	0.86
Weight (kg)	4.4 ± 1.9 *n*=21	4.2 ± 1.7 *n*=164	0.53
BSA (m^2)	0.26 ± 0.07 *n*=21	0.25 ± 0.07 *n*=156	0.75
Contrast total (mL)	31.8 ± 21.4 *n*=21	21 ± 18.1 *n*=144	0.013*
Contrast dose/BSA (mL/m^2)	123 ± 69.2 *n*=21	80.8 ± 56.2 *n*=138	0.002*
Contrast dose/weight (mL/kg)	7.56 ± 4.20 *n*=21	4.77 ± 3.27 *n*=143	0.001*
Creatine value prior to catheterization (mg/dL)	0.36 ± 0.17 *n*=19	0.46 ± 0.30 *n*=133	0.043*
Calculated GFR prior to catheterization (mL/min)	73.1 ± 30.9 *n*=19	71.2 ± 42.4 *n*=124	0.85
Creatine value after catheterization (mg/dL)	0.35 ± 0.11 *n*=18	0.45 ± 0.29 *n*=139	0.0091*
Calculated GFR after catheterization (mL/min)	68.5 ± 22.3 *n*=18	70.9 ± 40.8 *n*=128	0.71
Left ventricular fractional shortening (%)	40.7 ± 6.2 *n* = 8	35.8 ± 9.6 *n*=31	0.178
Left ventricular ejection fraction (%)	NA *n*=0	62.18 ± 8.03 *n*=32	NA

^*^P-value is significant

Patients with serosal and soft tissue enhancement received a higher mean total contrast volume (31.8±21.4 vs 21.0±18.1 mL; *P*-value=0.013), higher mean contrast volume per body weight (7.56±4.2 mL/kg vs 4.77±3.27 mL/kg; *P*-value=0.001), and higher contrast volume per body surface area (BSA) (123.0±69.2 vs 80.8±56.2 mL/m^2; *P*-value=0.002) than those without serosal and soft tissue enhancement. Serosal and soft tissue enhancement was not significantly correlated with BSA (*P*-value=0.69), weight (*P*-value=0.52), height (*P*-value=0.86), patient age (*P*-value=0.53), or sex (*P*-value=0.22).

**Fig. 3:**
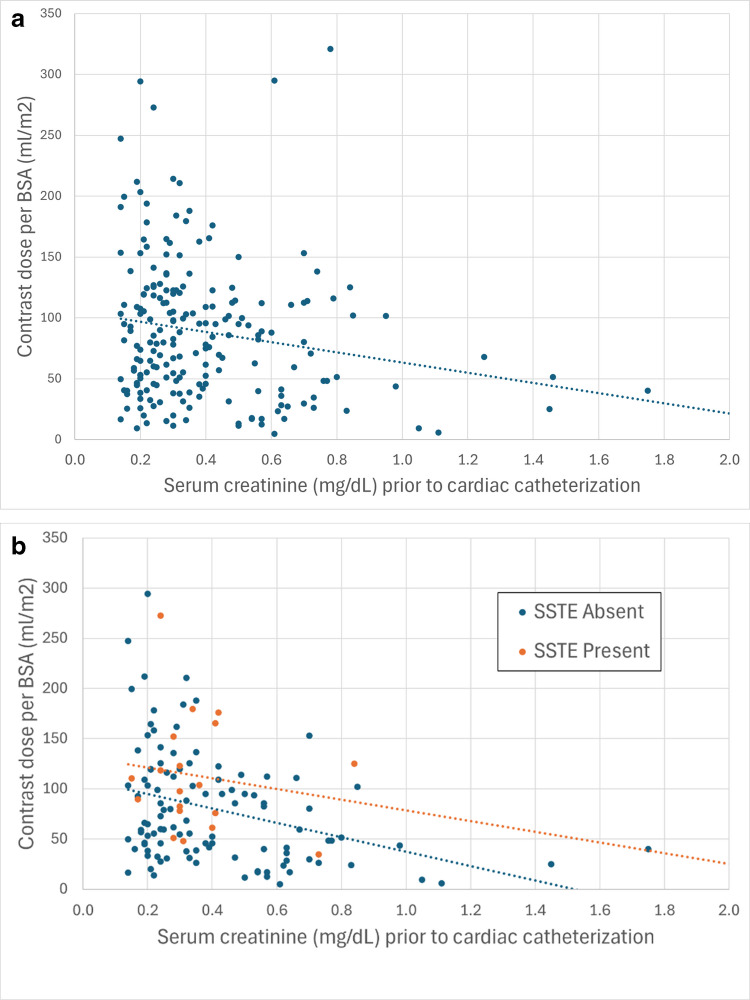
**a** Scatter diagram and fit plot showing that patients with lower pre-catheterization creatinine values received a significantly higher volume of intravenous contrast per body surface area (Pearson’s correlation coefficient *r*=−0.18; *P*=0.010). **b** Analysis of covariance scatter plot comparing pre-catheterization creatinine values with volume of intravenous contrast per body surface area shows no difference (*P*=0.81) between patients with (*orange circles* and *fit line*) and without (*blue circles* and *fit line*) a subsequent unanimous consensus diagnosis of serosal and soft tissue enhancement. Pearson’s correlation coefficients are *r*=−0.16 (95% CI: −0.57, 0.32) and *r*=−0.35 (95% CI: −0.50, −0.17) for patients with and without serosal and soft tissue enhancement, respectively

There was a significant correlation between lower pre-catheterization creatinine values and higher contrast volume per body surface area (Pearson’s correlation coefficient *r*=−0.18 with *P*-value=0.01). This relationship is shown in Fig. 3. When patients were stratified by the presence or absence of serosal and soft tissue enhancement, there was no significant difference between the two groups. The Pearson’s correlation coefficients are *r*=−0.16 (95% CI: −0.57, 0.32) and *r*=−0.35 (95% CI: −0.50, −0.17) for patients with and without serosal and soft tissue enhancement, respectively (*P*-value=0.81).

Cardiac physiology was reported for all 294 patients. Of this group, 125 patients (125/294; 42.5%) were reported to have a single ventricle physiology, while 169 (169/294; 57.5%) were reported to have a bi-ventricular physiology. When there was unanimous agreement among all reviewers regarding the presence or absence of serosal and soft tissue enhancement (Table 3), 115 patients had bi-ventricular physiology and 73 had a single ventricle physiology. Of these patients, those with a bi-ventricular physiology (17/115; 14.8%) had a significantly higher incidence of serosal and soft tissue enhancement compared to those with single ventricular physiology (4/73; 5.5%) with a *P*-value of 0.048.

**Table 3:** Association of ventricular physiology and a unanimous consensus diagnosis of serosal and soft tissue enhancement

	Number serosal and soft tissue enhancement present (%)	Number serosal and soft tissue enhancement absent (%)
Single ventricular physiology	4 (5.5)	69 (94.5)
Bi-ventricular physiology	17 (14.8)	98 (85.2)

Chi-square test shows a significant difference in the percentage of patients with bi-ventricular physiology with serosal and soft tissue enhancement compared to those with single ventricular physiology (*P*=0.048)

Left ventricular ejection fraction values were available for 41 patients in the study population. Of these, 32 (78.0%) were determined not to have serosal and soft tissue enhancement by unanimous consensus. No patients diagnosed with serosal and soft tissue enhancement had an ejection fraction reported. Left ventricular fractional shortening values were available for 61 patients in the study population. Of these, 31 (50.8%) did not have serosal and soft tissue enhancement, and 8 (13.1%) did. There was no significant difference in left ventricular fractional shortening between groups (40.7±6.2 vs 35.8±9.6; *P*-value=0.18).

To assess the impact of diastolic dysfunction on serosal and soft tissue enhancement, ventricular function was evaluated based on the ventricle receiving systemic venous return. Among bi-ventricular patients, diminished right ventricular function was present in 5 of 17 patients with serosal and soft tissue enhancement (29.4%) compared to 24 of 98 patients without enhancement (24.5%), a difference that was not statistically significant (*P*-value=0.18). In single ventricle patients, none of the four patients with serosal and soft tissue enhancement had abnormal ventricular function, whereas 17 of 69 patients without enhancement (24.6%) did (*P*-value=0.57). There were no significant differences in the frequency of serosal and soft tissue enhancement when ventricular dysfunction was further classified by severity (Table 4).

**Table 4:** Relationship between ventricular function and the presence or absence of serosal and soft tissue enhancement, stratified by single versus bi-ventricular physiology and by both the presence and severity of ventricular dysfunction

Comparison type		Serosal and soft tissue enhancement present	Serosal and soft tissue enhancement absent	*P*-value
Single ventricular function				
Presence of dysfunction	Normal	4 (100%)	52 (75.4%)	0.57
	Dysfunction present	0 (0%)	17 (24.6%)	
Severity of dysfunction	Normal	4 (19.1%)	52 (34.2%)	0.28
	Mild dysfunction	0 (0%)	12 (7.9%)	
	Moderate dysfunction	0 (0%)	4 (2.6%)	
	Severe dysfunction	0 (0%)	1 (0.7%)	
Bi-ventricular function				
Presence of dysfunction	Normal	12 (70.6%)	74 (75.5%)	0.76
	Dysfunction present	5 (29.4%)	24 (24.5%)	
Severity of dysfunction	Normal	12 (57.1%)	74 (44.9%)	0.16
	Mild dysfunction	2 (9.5%)	13 (7.9%)	
	Moderate dysfunction	2 (9.5%)	6 (3.6%)	
	Severe dysfunction	1 (4.8%)	5 (3.0%)	

## Discussion

Serosal and soft tissue enhancement is a unique imaging finding that has been reported to mimic pneumoperitoneum [1, 2]. In this study, we found that when assessed by unanimous agreement among three pediatric radiologists, serosal and soft tissue enhancement occurred in 7.1% of infants who underwent abdominal radiographs following cardiac catheterization. While the diagnosis remains somewhat subjective, this rate is higher than what has been suggested by prior case reports and indicates that the phenomenon may be more common than previously appreciated.

Several clinical factors were significantly associated with the presence of serosal and soft tissue enhancement, including lower pre- and post-catheterization creatinine levels, higher total contrast volume administered during the procedure, and higher contrast dose normalized to BSA and weight.

We believe that the contrast volume per BSA and contrast volume per weight are the most significant factors contributing to the development of serosal and soft tissue enhancement. Previous reports suggest that serosal and soft tissue enhancement results from increased lymphatic uptake of contrast agents, likely due to extracellular fluid redistribution driven by elevated hydrostatic pressure. This pressure increase can be further exacerbated by a state of volume overload, causing leakage of contrast and other fluids into the extracellular interstitium [1, 2]. Administering higher total doses of contrast, particularly when adjusted for BSA or weight, may amplify the volume overload, raise hydrostatic pressure, and promote greater retention of contrast within the tissues.

We initially hypothesized that the combination of decreased renal function and higher contrast dose would make the effect of soft tissue and serosal enhancement more pronounced due to decreased renal clearance of the contrast media. However, based on our data, a *lower* preprocedural creatinine level was associated with this phenomenon. The rationale for this finding is difficult to explain. To explain this finding, we further hypothesized that interventional cardiologists may be purposefully limiting contrast usage in patients with higher creatinine values. This hypothesis was also proven to be incorrect. While patients with a lower creatinine level prior to catheterization did receive more contrast per body surface area, there was no difference between groups in those who developed serosal and soft tissue enhancement and those who did not.

Currently, the diagnosis of serosal and soft tissue enhancement remains subjective. While all three reviewers agreed that serosal and soft tissue enhancement was present in 7.1% of children under 1 year of age who received an abdominal radiograph after cardiac catheterization, the incidence for individual reviewers ranged from 13.9 to 36.7%. This range highlights the subjective nature of the diagnosis and suggests that mild serosal and soft tissue enhancement may be present in more patients than reflected in the final consensus. We believe the reviewer with the highest detection rate may have been more attuned to subtle manifestations of this phenomenon, contributing to their higher identification rate. Given this variability, we recommend that radiologists maintain a high index of suspicion for this finding in infants who have recently undergone cardiac catheterization.

Key findings on abdominal radiograph include high density stranding within the subcutaneous fat, relative lucency of the liver shadow in relation to adjacent soft tissues, and enhancement of the pericardium and peritoneal surfaces. These findings occur while contrast remains visible within the urinary system. While there is little downside to missing subtle instances of this finding on radiograph, knowledge of the entity may help the radiologist explain an unusual appearance of a child’s soft tissues. For example, while we only explored this finding on abdominal radiograph, we have identified differing appearances on chest radiograph and thoracoabdominal CT.

While prior reports have suggested that soft tissue and serosal enhancement can mimic pneumoperitoneum, this diagnosis was not made clinically on any abdominal radiograph in patients determined to have soft tissue and serosal enhancement. However, in one patient, a diagnosis of pneumoperitoneum was made on a preceding chest radiograph and excluded by knowledge of the condition on the abdominal radiograph. Anecdotally, we have observed that this misdiagnosis is more common among trainees and on chest radiographs. The lack of misdiagnosis may reflect knowledge of the condition among our faculty as well as the study’s focus on abdominal radiographs. In patients with recent catheterization and continued imaging concern for pneumoperitoneum, additional imaging with a cross-table lateral or decubitus radiograph could be performed.

Serosal and soft tissue enhancement differs from other types of contrast opacification of soft tissues. Total body opacification of contrast is a distinct phenomenon characterized by widespread distribution of contrast media throughout the body’s tissues and organs following imaging procedures. Soft tissue and serosal enhancement typically occurs in infants after cardiac catheterization and shows a distinctive pattern: contrast enhancement appears in the peritoneum, pericardium, and soft tissues, while sparing solid organs. In contrast, total body opacification shows uniform enhancement across all body regions [3].

Different patterns of contrast enhancement, such as vicarious excretion, organ enhancement, and contrast retention, further illustrate the complexity of contrast media distribution. Vicarious excretion involves the non-renal excretion of contrast media, often seen in cases of renal impairment, leading to unusual enhancement in the liver and gastrointestinal tract [4–6]. Organ enhancement refers to specific uptake by various organs, indicating underlying pathologies like tumors or inflammation. Contrast retention occurs when the contrast media remains in the body tissues for extended periods due to impaired renal function, posing potential toxicity [7]. These patterns highlight the importance of understanding contrast media distribution for accurate diagnosis and patient management.

In pediatric imaging, the dosage of contrast media varies significantly between cardiac catheterization and CT scans. Cardiac catheterization generally involves higher total doses of contrast agents (1 mL/kg/bolus run) due to the need for detailed visualization of the heart and major vessels, which increases the risk of serosal and soft tissue enhancement and related phenomena. Contrast-enhanced CT scans typically use lower doses of contrast media (1-2 mL/kg/per scan) thereby reducing the likelihood of extensive contrast enhancement. In our study, patients who developed serosal and soft tissue enhancement received higher contrast doses during cardiac catheterization (7.56±4.20 mL/kg) compared to those without serosal and soft tissue enhancement (4.77±3.27 mL/kg), supporting the association between higher contrast exposure and the development of this phenomenon.

In evaluating cardiac physiology, we focused on the function of the ventricle receiving systemic venous return, based on the hypothesis that impaired ventricular function could reduce contrast clearance and contribute to the development of serosal and soft tissue enhancement. However, this hypothesis was not supported by our data, as no association was found between ventricular function and the presence of serosal and soft tissue enhancement. This finding is not unexpected, as other cardiac factors may play a role in the development of serosal and soft tissue enhancement, such as end-diastolic pressure and atrioventricular valve regurgitation. These variables represent important areas for future investigation.

There are several limitations of this study. Besides the primary limitation of identifying a subjective finding, the other main limitation is a potential referral bias. Of nearly 1800 patients who underwent cardiac catheterization, only approximately 1 in 6 underwent subsequent abdominal radiograph within the first 2 days after the procedure. It is possible that these patients were inherently different than the patients who did not receive abdominal radiograph. This difference may affect the frequency of diagnosis or the underlying risk factors. Additionally, it is possible that the inclusion time frame of a radiograph performed within 2 days of cardiac catheterization is not appropriate. A different time frame could influence the overall incidence of the finding. Finally, cardiac function was not determined for most patients. This limits our ability to determine if serosal and soft tissue enhancement is associated with ventricular function. However, there was no difference in left ventricular fractional shortening between patients with or without serosal and soft tissue enhancement.

## Conclusion

In conclusion, serosal and soft tissue enhancement occurs in a small proportion of neonates who undergo cardiac catheterization and is associated with higher dosages of iodine-based contrast and higher dosages per body surface area. Pediatric radiologists should be aware of this unique finding, which has been previously reported to mimic pneumoperitoneum.

## Data Availability

No datasets were generated or analysed during the current study.
